# Adalimumab in the management of refractory idiopathic retinal vasculitis, aneurysms, and neuroretinitis (IRVAN) syndrome: a case report and literature review

**DOI:** 10.1186/s12886-026-04692-1

**Published:** 2026-03-03

**Authors:** Ala Bassam Qadan, Mohammad Ibrahim Zafar, Mohammed Anass Tanveer, Bjarte Bondevik Berstad, Ragnhild Wivestad Jansson

**Affiliations:** 1 Faculty of Medicine, University of Bergen, Bergen, Norway; 2 Department of Ophthalmology, Haukeland University Hospital, Bergen, Norway; 3 Department of Clinical Medicine, Section of Ophthalmology, University of Bergen, Bergen, Norway

**Keywords:** IRVAN syndrome, Adalimumab, Tumour necrosis factor-alpha inhibitor, Retinal vasculitis, Macular oedema, Biologic therapy, Literature review, Case report

## Abstract

**Background:**

Idiopathic retinal vasculitis, aneurysms and neuroretinitis (IRVAN) is a rare ocular condition with an unresolved aetiology, different treatment practices and a risk of severe vision loss in refractory cases. There is sparse literature on the use of biologics, specifically TNF- α inhibitors, in the management of IRVAN, emphasising the need for additional evidence in this therapeutic area.

**Case presentation:**

We present a 49-year-old otherwise healthy Norwegian woman who experienced gradual bilateral visual deterioration and was diagnosed with IRVAN syndrome following extensive systemic and ophthalmological evaluation. Despite treatment with monthly bilateral intravitreal anti-vascular endothelial growth factor (anti-VEGF) injections, which were initially combined with systemic corticosteroids and later with peripheral scatter laser photocoagulation, disease control was not achieved. At nadir, the best-corrected visual acuity (BCVA) was 0.4 in OD and 0.3 in OS. Eight months after treatment initiation, the TNF-α inhibitor adalimumab was introduced in combination with methotrexate, and the subsequent clinical course was characterised by substantial and sustained improvement. The macroaneurysms regressed and the vasculitis subsided, leading to complete resolution of the peripapillary oedema and improvement of visual acuity to 0.9–1.0 bilaterally. At two years, the patient remained stable on adalimumab monotherapy without further intervention.

**Conclusions:**

Adalimumab may represent a safe and effective option for managing refractory IRVAN syndrome and could address the underlying pathology. Our findings underscore the importance of individualised therapeutic approaches and advocate further investigation of TNF-α inhibitors as a viable treatment option for IRVAN.

## Background

Idiopathic retinal vasculitis, aneurysms, and neuroretinitis (IRVAN) syndrome is an idiopathic inflammatory ocular disorder characterised by retinal vasculitis, multiple aneurysmal dilations of retinal arteries, and neuroretinitis [1]. It typically affects young, otherwise healthy individuals, and severe cases carry a significant risk of progressive vision loss if left untreated. The presumed pathogenesis involves immune-mediated vascular inflammation, leading to arterial wall weakening, macroaneurysm formation and vasculitis, with secondary retinal ischaemia, and neovascular complications [2].

Although neuroretinitis is part of the IRVAN acronym, Khairallah et al. argued that there is no clinical evidence of true neuroretinitis at any disease stage in patients with IRVAN [3]. They noted that optic disc staining on fluorescein angiography may occur, yet without functional features consistent with optic neuropathy or neuroretinitis and proposed that typical stellate or ring-shaped exudative maculopathy is driven primarily by leakage from retinal arteriolar aneurysms at or near the optic disc, which may be misinterpreted as neuroretinitis.

IRVAN is estimated to have a prevalence of less than 1 per 1,000,000 individuals [4]. Given its rarity, most of the existing knowledge is derived from case reports and small case series. The syndrome was first described by Kincaid and Schatz in 1983 and later defined more fully by Chang et al. in 1995, who proposed the acronym IRVAN after reporting a series of ten patients [1, 5]. Since then, only a few hundred cases have been documented in the literature.

Management strategies have historically included retinal laser photocoagulation, cryotherapy, vitrectomy surgery, systemic steroid treatment and injection of periocular or intravitreal steroids [6]. Later, intravitreal anti-vascular endothelial growth factor (anti-VEGF) therapy and steroid sparing disease-modifying antirheumatic drugs (DMARDs) have been added to the treatment options. However, treatment responses have been variable, and refractory cases remain a clinical challenge. Recently, anecdotal reports [7–14] have described the use of biologic agents such as tumour necrosis factor-alpha (TNF-α) inhibitors; however, evidence supporting their efficacy remains sparse. A thorough literature search, primarily conducted in PubMed, the Cochrane Library and Google Scholar, revealed only eight documented case reports describing the use of TNF-α inhibitors in IRVAN syndrome. Of these, only four involved the administration of adalimumab, in both early- and late-stage disease, underscoring the limited knowledge about treatment with biologics in this context.

We present a case of late-onset, treatment-refractory IRVAN syndrome, in which disease control was first achieved following administration of the TNF-α inhibitor adalimumab. This case adds to the limited body of literature and highlights the potential role of biologic therapy in managing refractory IRVAN. The observed clinical and anatomical improvements after treatment also provide a rationale for further investigation into targeted immunotherapy for this challenging condition. It may be of interest to explore earlier biologic intervention to reverse retinal vascular pathology and potentially prevent secondary ischaemic complications. A cytological profile in IRVAN has not been established, but TNF-α has been linked to the development of cerebral arterial aneurysms [15, 16].

## Case presentation– initial evaluation and early disease progression

A 49-year-old Norwegian woman with a family history of age-related macular degeneration presented with gradual bilateral visual decline over several months, which was more pronounced in the left eye. A few years earlier, she had a cutaneous melanoma of the right shoulder removed, but she was otherwise healthy, with no systemic symptoms.

**Fig. 1:**
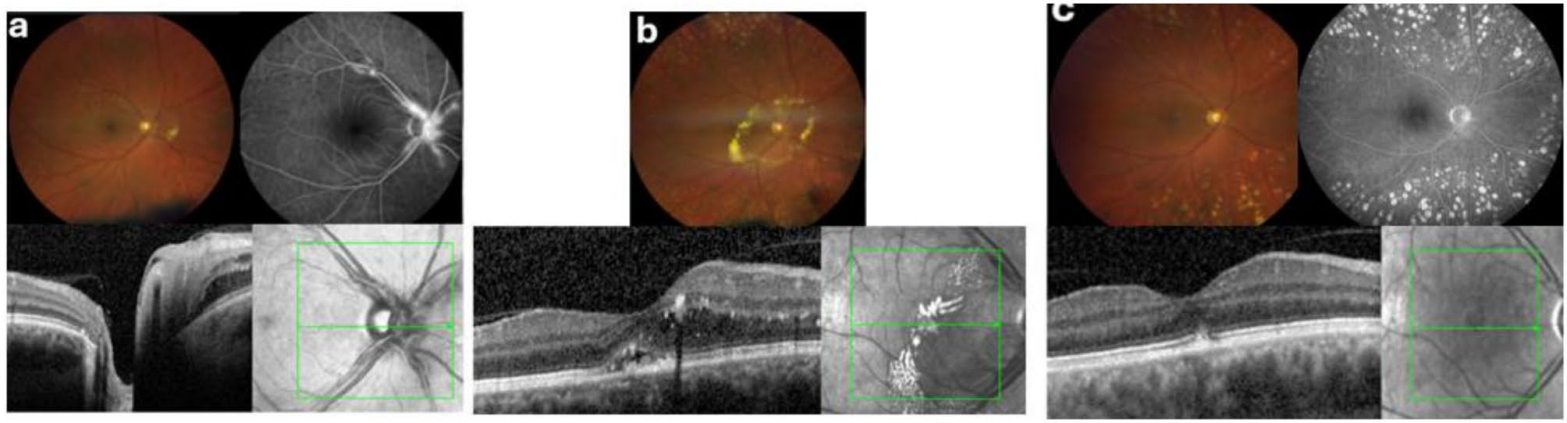
Sequential multimodal imaging of the right eye (OD). (**a**) Baseline — Month 0: Fundus photography, Fluorescein Angiography (FA) and Optical Coherence Tomography (OCT) before treatment showed vasculitis with peripheral sheating, proximal macroaneurysms (mainly at the disc) with leakage in late-phase FA and peripapillary haemorrhages and oedema with hard exudates nasal to the disc, but no macular oedema. (**b**) Pre-biologic — Month 19: Fundus photography and OCT after anti-VEGF and peripheral scatter laser treatment revealed persistent proximal macroaneurysms (some fibrosed) along with a marked peripapillary oedema encircled by a ring of hard exudates and involving the central macula. (**c**) Post-adalimumab — Month 40: Fundus photography demonstrated regressed macroaneurysms with complete resolution of edema and hard exudates, minimal macular pigment mottling, and absence of hemorrhages or neovascularization. FA showed quiescent vessels without leakage and only mild late staining of pre-papillary fibrotic changes. OCT confirmed a dry macula with preserved retinal architecture, intact foveal contour, and minimal RPE atrophy with pigment deposition

**Fig. 2:**
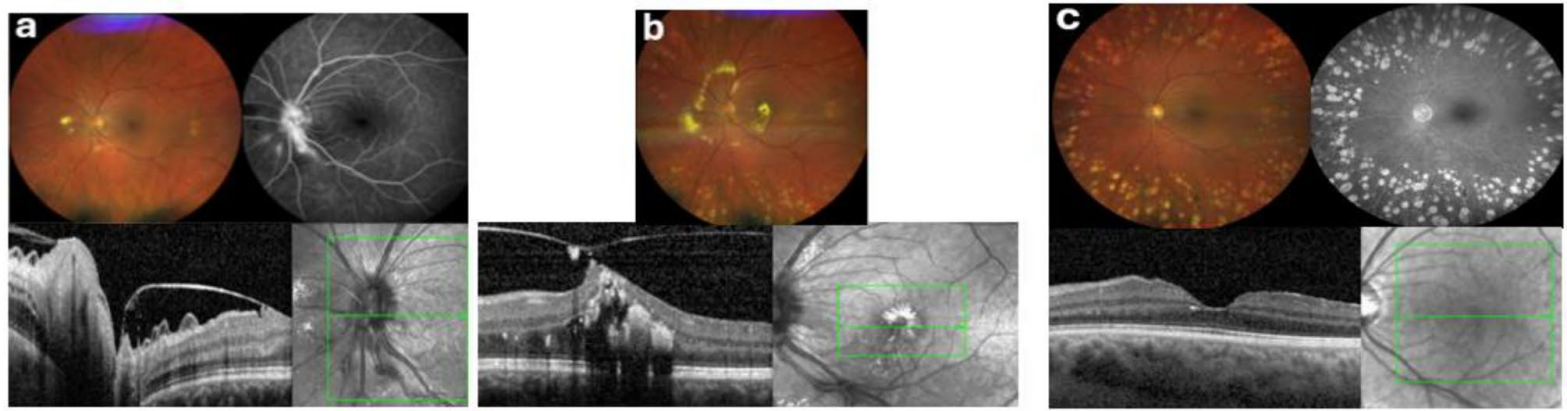
Sequential multimodal imaging of the left eye (OS). (**a**) Baseline — Month 0: Fundus photography and FA prior to treatment, showed vasculitis with peripheral sheating, proximal macroaneurysms showing late-phase leakage and peripapillary haemorrhages and oedema with hard exudates inferior and nasal to the disc. OCT demontrated ongoing posterior vitreous detachment with retinal traction, radial macular folds and peripapillary oedema. (**b**) Pre-biologic — Month 19: Fundus photography after anti-VEGF and peripheral scatter laser treatment revealed persistent proximal macroaneurysms (some fibrosed) along with a marked peripapillary oedema encircled by a ring of hard exudates and involving the central macula. OCT confirmed cystoid macular oedema with dense subfoveal hard exudates and marked vitreomacular traction with a small lamellar hole. (**c**) Post-adalimumab — Month 40: Fundus photography demonstrated regressed macroaneurysms with complete resolution of edema and hard exudates, some macular pigment mottling and pigment deposition, and absence of hemorrhages or neovascularization. FA showed quiescent vessels without leakage and only mild late staining of pre-papillary fibrotic changes. OCT confirmed a dry macula with preserved retinal architecture, intact foveal contour, complete posterior vitreous detachment and mild RPE atrophy with pigment deposition

Initial ophthalmological assessment showed Snellen best-corrected visual acuity (BCVA) of 1.0 in the right eye (OD) and 0.7 in the left eye (OS), with intraocular pressures of 20 mmHg OD and 15 mmHg OS. There was minimal anterior segment inflammation, no anterior segment neovascularisation, and mild vitritis. Fundus examination revealed bilateral retinal vasculitis, characterised by prominent proximal arteriolar macroaneurysms located at or adjacent to the optic disc, nerve fibre layer haemorrhages along the superior (OD) and inferior (OS) temporal arcades, and peripheral temporal arteriolar sheathing (Figs. 1a and 2a).

Fluorescein angiography (FA) demonstrated leakage from the arteriolar aneurysms, which were most prominent at the disc margin, along with delayed venous filling and inferotemporal retinal vessel narrowing in OS (Fig. 2a). At presentation, oedema and haemorrhages were confined nasal to the optic discs, with hard exudates at the nasal margin of the oedematous area. Over subsequent months, the peripapillary oedema gradually extended temporally and eventually included the central macula, delineated by a prominent ring of hard exudates (Figs. 1b and 2b).

Optical coherence tomography (OCT) confirmed progressive macular oedema and secondary vitreomacular traction in OS due to ongoing posterior vitreous detachment (Fig. 2b), correlating with visual decline. Widefield fluorescein angiography demonstrated peripheral capillary non-perfusion in both eyes, most pronounced temporally, without definite retinal or optic disc neovascularisation. However, detailed quantification of peripheral retinal capillary non-perfusion was not feasible because of suboptimal image quality in the far periphery.

### Diagnosis and pre-biologic treatment course

Given that retinal vasculitis with macroaneurysms and exudation can occur in several conditions, a comprehensive systemic and infectious evaluation was undertaken in addition to the multimodal retinal imaging. No evidence of connective tissue disease, ANCA-associated vasculitis, granulomatous inflammation, IgG4-related disease, antiphospholipid syndrome, cardiovascular disease, or infectious aetiologies was identified. The condition was therefore classified as isolated ocular vasculitis. Given the bilateral involvement and the combination of classical features, such as proximal arteriolar aneurysms including at bifurcations, retinal vasculitis, increasing peripapillary oedema with prominent hard exudates, and marked peripheral capillary non-perfusion, IRVAN syndrome was considered more likely than other differential conditions, such as senile arterial macroaneurysm, Coats’ disease, or Eales’ disease. Since the absence of neuroretinitis does not exclude IRVAN, the diagnosis of IRVAN syndrome was established 11 months after the initial presentation despite an atypically late disease onset. According to the IRVAN staging system, the angiographic features were consistent with stage 2 disease.

The patient was commenced on bilateral monthly intravitreal anti-VEGF injections (initially bevacizumab 1.25 mg/0.05 mL, later switched to aflibercept 2 mg/0.05 mL). Owing to worsening macular oedema and mild intraocular inflammation, high-dose oral corticosteroids (prednisolone 60 mg daily) were added and tapered over two months, resulting in partial oedema reduction but significant systemic side effects. On the basis of evidence supporting early laser treatment of non-perfused peripheral retina in IRVAN to prevent ischaemic complications, circumferential peripheral scatter laser photocoagulation (approximately 1800 spots OD, 1750 spots OS) was performed over a two-month period under continued monthly anti-VEGF therapy.

Despite intensive multimodal therapy, the patient experienced progressive visual deterioration with increasing macular oedema, vitreomacular traction, and persistent vascular leakage. BCVA reached a nadir of 0.4 OD and 0.3 OS. Treatment with monthly anti-VEGF injections was continued, and by the end of this treatment period BCVA reached up to 0.9 in OD and around 0.4 in OS, with reduced macular oedema; however, vasculitis and nasal peripapillary oedema remained.

Notably, BCVA began to improve towards the end of this period in OD; however, disease activity remained structurally evident, with persistent vasculitis, peripapillary oedema, and ongoing leakage despite repeated anti-VEGF, systemic corticosteroid, and peripheral scatter laser. Overall, the pre-biologic course was prolonged and characterised by slow, fluctuating functional changes without sustained structural resolution. Persistent structural activity necessitated an alternative therapeutic intervention. Consequently, immunomodulatory treatment with adalimumab and methotrexate was initiated approximately eight months after diagnosis.

### Clinical course after the initiation of adalimumab

Upon initiation of adalimumab 40 mg every two weeks with concurrent methotrexate (10 mg once per week) to mitigate immunogenicity, the patient reported subjective visual improvement within two months, confirmed by OCT findings demonstrating reduced intraretinal fluid and decreased macular oedema, particularly in OS. Methotrexate was discontinued shortly thereafter due to intolerable side effects, including fatigue. Anti-VEGF treatment was discontinued after two months, and adalimumab monotherapy was continued subcutaneously every two weeks and was well tolerated.

Over the subsequent 12–18 months, BCVA steadily improved and stabilised at 0.9–1.0 bilaterally. OCT imaging revealed complete resolution of macular oedema OD and significant reduction of exudation OS, with resorption of previously dense hard exudates. No further inflammation or neovascular complications were noted, and no additional intravitreal injections or laser interventions were needed. The arteriolar aneurysms regressed, and FA no longer showed leakage.

The patient continued regular follow-up every 2–3 months during the first year, extended to every 5–6 months thereafter. At nearly two years of follow-up, the patient remained clinically stable and in remission, with dry maculae, stable visual acuity, and no signs of active vasculitis. The tolerability of long-term adalimumab treatment remained excellent, and cautious tapering of the therapy was under consideration.

## Discussion and conclusion

This case describes the management of treatment-refractory IRVAN syndrome using adalimumab and aligns with other case reports describing the potential benefit of immunomodulatory therapy with TNF-α inhibitors in this disease. The findings also emphasise the possible role of TNF-α in the underlying disease mechanism. The favourable outcome documented here contributes to the limited literature on the use of TNF-α inhibitors in this disease.

Our case highlights two diagnostic considerations: the patient was older than typically reported in IRVAN, and the earliest manifestations were predominantly optic disc and peripapillary rather than a classic neuroretinitis phenotype. Nevertheless, the combination of characteristic retinal arterial macroaneurysms with vasculitis and peripheral non-perfusion, together with a negative systemic and infectious evaluation, made IRVAN the most plausible diagnosis in our patient.

Historically, IRVAN has been managed with systemic corticosteroids, laser photocoagulation, and anti-VEGF injections, although therapeutic responses have varied significantly, and refractory cases may continue to deteriorate despite aggressive intervention [2, 17]. This variability is reflected in several case reports, in which conventional therapies, including high-dose corticosteroids and photocoagulation, have yielded mixed and sometimes suboptimal outcomes [18–25].

In our presented case, the initiation of adalimumab was prompted by persistent inflammatory activity, macular oedema, and visual deterioration despite conventional treatment modalities. Treatment with adalimumab led to rapid clinical remission, as demonstrated by substantial visual acuity improvements, resolution of macular oedema, and control of vascular inflammation.

Our literature review identified eight published case reports describing the use of TNF-α inhibitors in IRVAN (Table 1). These cases included five females and five males, with a mean age at diagnosis of 21.2 years (range, 12–35 years). Treatment responses among these patients varied.

**Table 1:** Summary of demographics, clinical presentation, treatment and outcomes in reported cases IRVAN treated with TNF-α inhibitors

Reference	Age (yrs) /Race/ Gender	Eye	Initial VA	Final VA	Initial Stage*	Worst Stage*	Initial Presentation	Clinical Findings	Treatment Strategy	Therapy Response	Follow up (mo)	Comments
Cheema et al. [7]	29/ME/M	Right Left	20/80 20/20	20/20 20/20	2 2	3 2	Floaters, blurred vision	**OU**: mild anterior-chamber cells & vitritis; extensive peripheral CNP; vessel sclerosis; no NV at baseline **OD**: optic-disc hyperaemia; macular oedema + exudation; aneurysmal arterioles; later peripheral NV. **OS**: optic-disc hyperaemia; no macular oedema or NV.	6 weeks of prednisolone 80 mg daily was followed by infliximab 5 mg/kg IV (administered at weeks 0,4,8 and 12, then q2 mo); PRP was performed upon NV appeared.	No response to prednisolone. Dramatic resolution following infliximab. Inflammation and exudates resolved; VA improved to 20/20; NV regressed after PRP; quiescent on 3-monthly infliximab	18	First IRVAN cases on infliximab
	35/ME/F	Right Left	20/20 20/40	20/20 20/20	2 3	3 3	Floaters	**OU**: moderate vitritis; extensive peripheral CNP; floaters; systemic work-up negative **OD**: single aneurysmal arteriole near disc; no NV. **OS**: optic-disc hyperaemia + disc NV; macular oedema + exudation; mild VH; VA dropped to 20/40 at 6 wk	Prednisolone 60 mg daily + PRP OU; mycophenolate 1 g daily; infliximab single loading dose followed by full IV schedule (0-4-8-12 wk, then q2 mo)	Disc leakage & macular oedema resolved; VA improved to 20/20 OU; disease quiescent on maintenance infliximab	24	
Mansour et al. [8]	30/ME/F	Right Left	20/20 20/20	20/20 CF 1 m	0 / N/A 2	2 3	Floater in OS	**OU**: IOP 8 mm Hg; +1 anterior-chamber cells; moderate vitritis; extensive peripheral CNP; no systemic disease. **OD**: initially normal; 2 y later branch-artery leak with macular edema and peripheral ischemia; macro-aneurysms on FFA. **OS**: retinal vasculitis, optic-disc hyperemia; wide CNP with disc leakage; macro-aneurysms; macular edema; mild VH; later RPE detachment followed by RPE rip and subsequent macular scar	6 mo oral prednisolone + mycophenolate 500 mg qd (no response), followed by infliximab, limited PRP, bevacizumab, and repeated aflibercept for 6 mo (no response), after which heavy PRP was performed in both eyes (stage II/III)	All retinal leaks resolved; RE edema subsided, VA remained 20/20; OS progressed to macular scar, final VA counting-fingers 1 m; stable on observation; no further procedures after heavy PRP	96	Heavy PRP effective at stages II–III; delayed diagnosis left OS with poor vision; underscores need for early IRVAN detection. Stage-0 note: OD was clinically normal at presentation so listed as stage 0 (outside IRVAN spectrum) before later progression.
Samalia et al. [9]	16/C/M	Right Left	20/50 CF	20/25 20/60	2 2	2 2	Bilateral progressive visual impairment	**OU**: Fundus: Neuroretinitis; macular and peripapillary exudates; retinal vasculitis; FFA: Disc leakage; peripheral CNP, ICG: peripapillary aneurysmal dilations; OCT: Macular exudates **OS**: OCT: Subfoveal fluid	Doxycycline 100 mg BID, rifampicin 300 mg BID, and oral prednisone 1 mg/kg were initiated for presumed Bartonella neuroretinitis. Due to lack of response, treatment was switched to monthly intravenous infliximab 5 mg/kg	Marked improvement in vasculitis and aneurysms was observed within 3 mo. OD: macular exudates resolved; OS: subfoveal fluid resolved	≥ 3	Demonstrated poor response to high-dose oral corticosteroids, with persistent vasculitis and aneurysms necessitating initiation of infliximab
	14/SA/M	Right Left	20/40 20/30	20/20 20/20	2 2	2 2	Progressive visual decline. Retinal vascular changes, macular exudation and aneurysmal changes	**OU**: FFA: peripheral CNP; ICG: peripapillary aneurysmal dilations **OD**: macular exudation **OS**: peripapillary exudation	IV methylprednisolone (3 × 100 mg pulses) followed by oral prednisone taper (80 mg to 5 mg); infliximab IV 5 mg/kg initiated within 4 weeks; FFA-guided PRP OU for peripheral ischaemia	Aneurysms and vasculitis resolved within 6 weeks; exudate reduction observed; peripheral retinal ischaemia improved angiographically	≥ 1.5	Early initiation of infliximab in combination with corticosteroids and laser therapy led to rapid anatomical improvement and disease stabilization
Singh et al. [10]	13/SA/F	Right Left	20/80 20/80	20/40 20/60	1 1	1 1	Central scotoma	**OU**: Neuroretinitis; 1 + vitreous cells; disc & arcade aneurysms; macular edema wit hard exudates; active vasculitis; OCT: subfoveal exudates; FFA: aneurysmal leakage, no CNP	Dexamethasone intravitreal implants OU with oral MMF 1 mg BID (transient response); after 6 months, switched to Adalimumab 20 mg SC q2w + Azathioprine (titrated to 50 mg BID); Adalimumab later increased to 40 mg SC q2w	Treatment resulted in significant reduction of inflammation, macular exudation, and aneurysm size, with no evidence of CNP on FFA. Disease remained clinically controlled	15	First reported IRVAN case treated with Adalimumab in combination with Azathioprine: with no systemic side effects observed
Klon et al. [11]	19/C/F	Right Left	20/32 20/20	NA NA	2 3	2 3	Visual deterioration in the right eye, with a known history of retinal vasculitis	**OU**: peripapillary hard exudates; blurred disc margins, arteriolar aneurysmal dilations (around disc and arcades); perivascular sheathing; segmental vascular occlusions; dot-blot hemorrhages; OCT; no exudative activity; FFA: peripheral CNP; segmental vascular leakage **OD**: macular hard exudates. **OS**: FFA; NVE.	IV prednisolone 100 mg/day for 3 days, followed by oral taper (80 mg reduced to 5 mg); no clinical improvement. Adalimumab 40 mg SC q2w for 3 mo. (overlapping with low dose prednisolone). PRP to both eyes for peripheral ischemia. OS: Persistent neovascularization treated with intravitreal bevacizumab and additional laser photocoagulation	Adalimumab led to reduced vascular leakage and peripapillary exudates; however, NVE in the left eye progressed initially. After adjunctive intravitreal bevacizumab and additional laser, NVE regressed, and inflammation resolved. No relapse to date under close monitoring.	≥ 3	Combination therapy showed partial response; left eye NVE regressed only after anti-VEGF
Joy Li et al. [13]	24/NA/M	Right Left	20/40 20/30	20/25 − 20/30 20/25 − 20/30	2 2	3 3	Bilateral photopsia followed by sequential nasal paracentral scotomas	**OU**: retinal vasculitis, macroaneurysms, peripheral nonperfusion, vascular sheating, disck leakage, vitreous cells **OD**: vessel pruning abutting temporal fovea **OS**: more severe vessel pruning, vitreous hemorrhage (late), required vitrectomy	Oral prednisone 100 mg qd for 1 week, followed by IV methylpredinosolone 1 g/day for 3days, then tapered to 60 mg; started on Adalimumab, subtenon triamcinolone OS; intravitreal bevacizumab OU; transitioned to Rituximab and MMF (1 g BID), later switched to Infliximab and MMF (1.5 g BID); PRP OU; vitrectomy OS	Disease stabilized with infliximab + MMF; vessel reperfusion by 6 mo; stable vision after 28 mo.	28	Refractory to steroids, adalimumab, and rituximab; disease stabilized with infliximab-MMF; OS vitrectomy required for vitreous hemorrhage
Mandura et al. [14]	12/NA/F	Right Left	20/20 20/20	20/20 20/20	2 2	2 2	Bilateral floaters	**OU**: optic disc swelling, peripapillary and macular hard exudates, aneurysmal dilations (disc and arterioles), vascular sheating, peripheral ischemia; OCT: no macular edema; FFA: inflamed optic discs with aneurysmal leakage and peripheral nonperfusion; no NV	Oral prednisone 1 mg/kg daily, MMF 1 g BID, followed by PRP OU (no response); transitioned to infliximab IV 5 mg/kg at weeks 0, 2, and 6, then every 6 weeks x3, every 8 weeks x4, every 10 weeks x3, and every 12 weeks x3	Complete resolution of vasculitis, aneurysms, optic disc swelling, and macular exduates within 6 mo.; stable remission maintained through 36 mo.	36	Inflammation was unresponsive to corticosteroids and MMF; sustained disease control achieved with infliximab monotherapy
Van Winckel et al. [12]	20/NA/M	Right Left	20/22 20/67	NA NA	2 2	2 3	Floaters and reduced vision	**OU**: retinal vasculitis; peripheral non-perfusion; aneurysmal dilations **OD**: NV, vitreous hemorrhage. **OS**: capillary dropout, aneurysms, initial vitreous hemorrhage	Oral prednisone (unspecified dose), PRP OU, multiple anti-VEGF (7x OD, 6x OS); due to persistent inflammation, switched to Adalimumab (80 mg day 1, followed by 40 mg qw then q2w	Marked reduction in vasculitis, aneurysms, and peripheral ischemia; OS NVE regressed; partial reperfusion on FA; anatomy remained stable under Adalimumab	≥ 18	Adalimumab achieved anatomical control in steroid- and anti-VEGF-refractory IRVAN

BID = bis in die, twice a day; C = Caucasian; CF = Counting fingers; CNP = Capillary non-perfusion; F = female; FFA = Fundus Fluorescein Angiography; ICG = Indocyanine green angiography; IOP = intra-ocular pressure; M = male, ME = Middle Eastern; MMF = Mycophenolate Mofetil; NA = Not Available; NV = neovascularization; NVD = Neovascularization of the Disc; NVE = Neovascularization Elsewhere; OCT = Optical Coherence Tomography; OD / OS / OU = right eye / left eye / both eyes; PRP = panretinal photocoagulation · RPE = retinal pigment epithelium; SA = South Asian; SC = subcutaneous; VA = visual acuity; VH = vitreous haemorrhage; qd = every day; q2 mo = every 2 months; q2w = once every 2 weeks; qw = weekly

*IRVAN Staging (Samuel et al.,* 2007)*:

**Stage 1** – macroaneurysms;

**Stage 2** – + peripheral capillary non-perfusion;

**Stage 3** – + neovascularization of posterior segment;

**Stage 4** – + anterior segment neovascularization;

**Stage 5** – + neovascular glaucoma

Singh et al. described significant clinical improvement and inflammation control with adalimumab combined with azathioprine, highlighting the possible efficacy of this combination in IRVAN management [10]. Van Winckel et al. reported quiescence of vasculitis after prior panretinal photocoagulation and anti-VEGF, with angiographic improvement and partial reversal of capillary non-perfusion over 9–18 months. This finding suggests that timely TNF-α blockade may permit microvascular reperfusion where occlusion is predominantly inflammatory, whereas ischaemia-driven sequelae still require PRP or anti-VEGF. In contrast, Klon et al. reported that adalimumab alone was insufficient to manage persistent neovascularisation, requiring additional anti-VEGF therapy to achieve disease stabilisation [11].

Cheema et al. demonstrated substantial clinical improvement with infliximab following initial treatment failure with corticosteroids, noting rapid resolution of inflammatory signs, neovascularisation, and improvement in visual acuity [7]. Samalia et al. described favourable outcomes when infliximab was initiated early, with notable resolution of aneurysms and retinal vasculitis [9]. Joy Li et al. described a refractory case that progressed despite the use of corticosteroids, adalimumab, and rituximab, but stabilised after switching to infliximab combined with mycophenolate mofetil [13]. Areas of vascular reperfusion were documented, yet neovascular complications still needed targeted PRP and intravitreal anti-VEGF, underscoring the roles of class-switching within anti–TNF-α and combination immunomodulation alongside ischaemia-directed therapy [13]. Similarly, Mandura et al. reported complete resolution of vasculitis, aneurysms and optic disc swelling within six months of infliximab initiation in a paediatric patient who was unresponsive to corticosteroids and mycophenolate mofetil. Disease remission was sustained for 36 months, underscoring the potential for long-term efficacy of infliximab in IRVAN treatment [14]. Conversely, Mansour et al. reported a patient with no significant therapeutic benefit from infliximab, who ultimately required heavy laser photocoagulation to achieve disease stabilisation [8].

The discrepancies among these case reports highlight the complexity and heterogeneity of IRVAN syndrome, suggesting that the therapeutic response may be influenced by patient-specific factors, disease severity, and the timing of intervention. Accordingly, TNF-α inhibitors should be considered with caution, individualised to patients, and closely monitored.

The strengths of this case report include detailed longitudinal documentation of the therapeutic response and thorough systemic evaluation excluding confounding conditions. The atypical age at presentation also broadens the known demographic spectrum of IRVAN and reinforces the diagnostic relevance of this case.

Limitations include limited generalisability, lack of a control group, and potential observer bias. Several pre-biologic therapies overlapped, and BCVA in the right eye had begun to improve before adalimumab, so delayed effects of prior treatments and/or natural disease fluctuations may have contributed. Nevertheless, the pre-biologic period was prolonged (> 8 months) and marked by slow, fluctuating functional changes without sustained structural resolution, whereas after adalimumab initiation the course shifted towards more rapid and sustained bilateral quiescence. The counterfactual course remains unknown; therefore, our findings support an association rather than proof of efficacy. Even so, the temporal link between adalimumab initiation and sustained improvement provides supportive, although not definitive, evidence that TNF-α inhibition may be beneficial in selected refractory IRVAN patients. Finally, heterogeneity in patient characteristics and prior treatments across the literature limits firm conclusions about the broader applicability of adalimumab or other biologic agents.

Nevertheless, our findings reinforce the hypothesis that targeted biologic therapies, particularly adalimumab, may modulate the immune-mediated pathology of IRVAN effectively in specific patient populations. The sustained clinical stability and tolerability observed in our patient over a nearly two-year treatment period align with previous reports by Singh et al. However, additional research is necessary to establish definitive safety profiles and optimal treatment durations.

Given the rarity and clinical heterogeneity of IRVAN, larger prospective studies and multicentre collaborations can provide more comprehensive insights into optimal therapeutic strategies and long-term outcomes. Future research should focus on refining patient selection criteria, identifying the appropriate timing for treatment initiation, and systemically evaluating the long-term safety and efficacy of biological treatments.

In conclusion, this case illustrates the potential therapeutic benefit of adalimumab in refractory IRVAN syndrome, suggesting its role in targeting underlying inflammatory mechanisms and adding to the limited body of evidence on biologic therapy in this condition. These findings emphasise the need for individualised treatment strategies and support further investigation into the early use of biologics to mitigate ischaemic complications associated with severe disease.

## Data Availability

No datasets were generated or analysed during the current study.

## Abbreviations

AAV: ANCA-associated vasculitis
ANCA: Anti neutrophil cytoplasmic antibody
BCVA: Best corrected visual acuity
CMV: Cytomegalovirus
FA: Fluorescein angiography
IgG4: Immunoglobulin G4
ILM: Internal limiting membrane
IRVAN: Idiopathic retinal vasculitis, aneurysms, and neuroretinitis
OCT: Optical coherence tomography
OD: Oculus dexter (right eye)
OS: Oculus sinister (left eye)
TNF-α: Tumour cecrosis factor-alpha
VEGF: Vascular endothelial growth factor
